# 髓外病变对初诊多发性骨髓瘤患者预后的影响

**DOI:** 10.3760/cma.j.issn.0253-2727.2023.01.009

**Published:** 2023-01

**Authors:** 怡 陶, 诗炜 金, 焰 王, 思洁 唐, 元昉 刘, 捷 许, 萌萌 潘, 卫平 章, 坚青 糜

**Affiliations:** 上海血液学研究所，医学基因组学国家重点实验室，国家转化医学中心（上海），上海交通大学医学院附属瑞金医院，上海 200025 Shanghai Institute of Hematology, State Key Laboratory of Medical Genomics, National Research Center for Translational Medicine at Shanghai, Ruijin Hospital Affiliated to Shanghai Jiao Tong University School of Medicine, Shanghai 200025, China

**Keywords:** 多发性骨髓瘤, 髓外病变, 骨旁髓外, 软组织髓外, 自体造血干细胞移植, 预后, Multiple myeloma, Extramedullary disease, Bone-related extramedullary disease, Extraosseous extramedullary disease, Autologous hematopoietic stem cell transplantation, Prognosis

## Abstract

**目的:**

比较初诊多发性骨髓瘤（NDMM）非髓外病变（non-EMD）、骨旁髓外（EM-B）和软组织髓外（EM-E）患者的临床特征及预后，并探讨自体造血干细胞移植（ASCT）对EMD的影响。

**方法:**

回顾性分析上海交通大学医学院附属瑞金医院2015年1月至2022年1月收治的515例NDMM患者，114例（22％）伴EMD患者，其中EM-B组91例（18％），EM-E组23例（4％）。通过卡方检验比较3组患者临床特征的差异；通过Kaplan-Meier法比较3组患者无进展生存（PFS）和总生存（OS）差异；通过Cox比例风险模型的多因素分析确定独立预后因素。

**结果:**

三组患者在年龄、性别、国际分期系统（ISS）分期、轻链、肌酐清除率、细胞遗传学危险度、17p缺失、是否接受ASCT和诱导方案类型等方面差异均无统计学意义。13％ EM-E患者M蛋白为IgD型，显著增高（*P*＝0.021）。non-EMD、EM-B和EM-E 3组患者中位PFS时间分别为27.4、23.1和14.0个月，中位OS期分别为未达到、76.8个月和25.6个月，EM-E的PFS（对non-EMD，*P*＝0.004；对EM-B，*P*＝0.036）和OS（对non-EMD，*P*<0.001；对EM-B，*P*＝0.002）最差，而EM-B与non-EMD组之间PFS和OS差异无统计学意义。多因素分析中，EM-E是NDMM患者OS的独立预后危险因素（*HR*＝8.779，*P*<0.001），对PFS也产生不良影响（*HR*＝1.874，*P*＝0.050）。在未接受ASCT患者中，EM-B患者OS显著差于non-EMD组（中位76.8个月对未达到，*P*＝0.029），但在ASCT患者中，EM-B和non-EMD组PFS和OS差异无统计学意义。

**结论:**

与初诊non-EMD、EM-B比较，EM-E患者预后最差，而且EM-E是NDMM患者OS的独立危险因素。ASCT能克服EM-B的不良预后。

## Abstract (en)

**Objective:**

To summarize the characteristics of patients with newly diagnosed multiple myeloma（NDMM）admitted at Ruijin Hospital affiliated to Shanghai Jiaotong University School of Medicine. We compared the clinical characteristics and prognoses among patients with non-extramedullary disease（EMD）, bone-related extramedullary（EM-B）disease, and extraosseous extramedullary（EM-E）disease and further explored the effects of autologous hematopoietic stem cell transplantation（ASCT）for EMD.

**Methods:**

From January 2015 to January 2022, data of 114 patients（22％）with EMD out of 515 patients with NDMM were retrospectively analyzed; 91（18％）and 23（4％）patients comprised the EM-B and EM-E groups, respectively. The clinical characteristics of patients in all groups were compared with the Chi-square test. Progression-free survival（PFS）and overall survival（OS）of patients were analyzed by the Kaplan-Meier method. Independent prognostic factors were determined using multivariate Cox proportional hazard model.

**Results:**

There were no significant differences in age, gender, ISS stage, light chain, creatinine clearance, cytogenetic risk, 17p deletion, ASCT, and induction regimens among the three groups. Overall, 13％ of EM-E patients had IgD-type M protein, which was significantly higher than that in EM-B patients（*P*＝0.021）. The median PFS of patients in the non-EMD, EM-B, and EM-E groups was 27.4, 23.1, and 14.0 months; the median OS was not reached, 76.8 months, and 25.6 months, respectively. The PFS（*vs* non-EMD, *P*＝0.004; *vs* EM-B, *P*＝0.036）and OS（*vs* non-EMD, *P*<0.001; *vs* EM-B, *P*＝0.002）were significantly worse in patients with EM-E, while those were not significantly different between patients with EM-B and those with non-EMD. In the multivariate analysis, EM-E was an independent prognostic factor for OS in patients with NDMM（*HR*＝8.779, *P*<0.001）and negatively impacted PFS（*HR*＝1.874, *P*＝0.050）. In those who did not undergo ASCT, patients with EM-B had significantly worse OS than those with non-EMD（median 76.8 months *vs*. not reached, *P*＝0.029）. However, no significant difference was observed in the PFS and OS of patients with EM-B and those with non-EMD who underwent ASCT.

**Conclusion:**

Compared to patients with either non-EMD or EM-B, those with EM-E had the worst prognosis. EM-E was an independent risk factor for OS in patients with NDMM. ASCT can overcome the poor prognosis of EM-B.

髓外病变（EMD）是多发性骨髓瘤（MM）的常见表现，其特点是骨髓瘤细胞能够在缺乏骨髓微环境的情况下生存和增殖[1]。根据EMD发生时间，分为初诊EMD和在MM疾病进展或复发时的继发EMD。根据EMD位置，分为骨旁髓外（EM-B）病变和软组织髓外（EM-E）病变。EM-B易累及中轴骨（肋骨、椎骨、颅骨、胸骨和骨盆）；而EM-E易累及皮肤、肝脏、中枢神经系统、胸腔积液、肾脏、淋巴结和胰腺等[2]。初诊和继发EMD、EM-B和EM-E预后各不相同，本文我们主要讨论初诊EMD，并区分EM-B和EM-E。作为MM治疗一线地位的自体造血干细胞移植（ASCT）对初诊EMD的影响，以及初诊EMD能否作为独立的预后因素，目前仍不清楚。我们通过回顾性分析515例初诊多发性骨髓瘤（NDMM）患者，比较初诊非髓外病变（non-EMD）、EM-B和EM-E患者临床特征和预后，初步探索上述问题。

## 病例与方法

一、病例

回顾性分析上海交通大学医学院附属瑞金医院2015年1月至2022年1月收治的515例NDMM患者，其中伴EMD患者共114例，包括EM-B 91例，EM-E 23例（表1）。EMD定义为在符合MM诊断标准基础上，同时伴浆细胞瘤骨髓外浸润，其中突破骨皮质侵犯周围软组织者定义为EM-B，由血源性播散引起远离骨的解剖部位发生软组织肿瘤者定义为EM-E，若患者同时存在EM-B和EM-E，则归为EM-E组。本研究经我院伦理委员会批准，并获得患者的知情同意。

**表1:** 515例NDMM患者中non-EMD、EM-B和EM-E三组患者临床特征比较

临床特征	non-EMD（401例）	EM-B（91例）	EM-E（23例）	*χ*^2^值	*P*值
年龄［例（％）］				4.410	0.110
>65岁	148（36.9）	25（27.5）	11（47.8）		
≤65岁	253（63.1）	66（72.5）	12（52.2）		
性别［例（％）］				2.456	0.293
男	232（57.9）	56（61.5）	10（43.5）		
女	169（42.1）	35（38.5）	13（56.5）		
ISS分期［例（％）］				3.760	0.153
Ⅰ~Ⅱ期	296（74.2）	69（75.8）	13（56.5）		
Ⅲ期	103（25.8）	22（24.2）	10（43.5）		
M蛋类型［例（％）］				6.895	0.142
轻链	84（20.9）	13（14.3）	6（26.1）		
重链+轻链	312（77.8）	74（81.3）	17（73.9）		
未分泌型	5（1.2）	4（4.4）	0（0.0）		
M蛋白重链［例（％）］				7.766	0.021
IgD型	14（3.5）	1（1.1）	3（13.0）		
非IgD型	387（96.5）	90（98.9）	20（87.0）		
肌酐清除率［例（％）］				1.906	0.386
<60 ml/min/1.73 m^2^	104（26.1）	25（27.5）	9（39.1）		
≥60 ml/min/1.73 m^2^	295（73.9）	66（72.5）	14（60.9）		
细胞遗传学危险度［例（％）］				0.881	0.644
标危	225（75.0）	57（80.3）	13（76.5）		
高危	75（25.0）	14（19.7）	4（23.5）		
17p缺失［例（％）］				3.012	0.222
是	35（11.5）	6（8.6）	4（23.5）		
否	270（88.5）	64（91.4）	13（76.5）		
接受ASCT［例（％）］				5.468	0.065
是	129（32.2）	35（38.5）	3（13.0）		
否	272（67.8）	56（61.5）	20（87.0）		
诱导方案［例（％）］				9.797	0.133
PI为主	311（77.6）	72（79.1）	17（73.9）		
IMiD为主	46（11.5）	4（4.4）	1（4.3）		
PI+IMiD为主	38（9.5）	14（15.4）	5（21.7）		

注 NDMM：初诊多发性骨髓瘤；non-EMD：非髓外病变；EM-B：骨旁髓外病变；EM-E：软组织髓外病变；ISS分期：国际分期系统；ASCT：自体造血干细胞移植；PI：蛋白酶体抑制剂；IMiD：免疫调节剂

二、方法

1. 诊断及疗效评估：收集515例患者初诊时外周血和骨髓标本，其中392例（76％）患者进行了荧光原位杂交（FISH）检查，骨髓标本经CD138单克隆抗体磁珠分选，分别检测了17p缺失、1q21扩增、13q14缺失、IgH重排，对有IgH重排的患者，进行t（4;14）、t（11;14）、t（14;16）和t（14;20）次筛。高危细胞遗传学按照修订的国际分期系统（R-ISS）分期标准[3]定义为17p缺失、t（4;14）、t（14;16）。因4例患者次筛标本不足，最终388例患者进行高危/标危细胞遗传学分类。114例EMD患者的影像学检查包括PET-CT 95例（83％）、CT 14例（12％）、MRI 5例（5％）。61例（54％）EMD患者进行髓外病灶的病理活检及免疫组化，同时排除孤立性骨浆细胞瘤、孤立性髓外浆细胞瘤和浆细胞白血病。MM的诊断和疗效评估参照国际骨髓瘤工作组标准[4]。髓外病变的大小以测量肿块的最大长径为代表。

2. 治疗方案：515例患者中508例接受以新药为基础的三药联合方案进行诱导化疗，7例使用传统化疗药物（包括长春碱类、阿霉素、环磷酰胺）联合激素方案治疗。新药包括蛋白酶体抑制剂（PI）硼替佐米、伊沙佐米、卡非佐米和免疫调节剂（IMiD）来那度胺。其中400例患者使用PI为基础的化疗（377例硼替佐米+20例伊沙佐米+3例卡非佐米），而一线使用伊沙佐米或卡非佐米的患者参加相应临床试验；51例患者使用IMiD来那度胺为基础的治疗；57例患者使用联合PI和IMiD为基础的方案。167例患者接受ASCT，其中non-EMD 129例、EM-B 35例、EM-E 3例。114例EMD患者中21例在化疗前后进行干预，按干预方式分为：单纯手术11例、手术联合放疗6例、单纯放疗4例；按病变位置分：EM-B 19例，EM-E 2例。患者复发后选用诱导治疗未使用的其他新药。

3. 随访：通过查阅门诊或住院病历及电话进行随访，随访截至2022年3月31日，中位随访18.1（95％*CI* 16.3～19.8）个月。无进展生存（PFS）时间定义为患者启动一线治疗至疾病进展或死亡的时间。总生存（OS）时间定义为患者启动一线治疗至死亡或末次随访的时间。

4. 统计学处理：采用SPSS 25.0和Graphpad Prism 7.0软件进行统计学分析。患者临床特征的比较采用*χ*^2^检验或Fisher精确概率法，采用Kaplan-Meier法绘制生存曲线，Log-rank检验进行组间比较。多因素分析采用Cox比例风险模型。双侧*P*<0.05为差异有统计学意义。

## 结果

1. non-EMD、EM-B和EM-E三组患者临床特征比较：515例NDMM患者中，EMD占22％，包括18％ EM-B和4％ EM-E。non-EMD、EM-B以及EM-E三组患者在年龄、性别、ISS分期、是否轻链、肌酐清除率、细胞遗传学危险度、17p缺失、是否接受ASCT和诱导方案类型等差异均无统计学意义。13％ EM-E患者M蛋白为IgD型，显著增高（*P*＝0.021）。

2. 预后分析：通过对整体NDMM患者人群分析，non-EMD、EM-B和EM-E三组患者中位PFS期分别为（27.4±1.8）个月、（23.1±3.5）个月及（14.0±1.9）个月（三组总体*χ*^2^＝8.758，*P*＝0.012；EM-B对non-EMD，*χ*^2^＝1.142，*P*＝0.285；EM-E对non-EMD，*χ*^2^＝8.153，*P*＝0.004；EM-B对EM-E，*χ*^2^＝4.398，*P*＝0.036）（图1A）。non-EMD、EM-B和EM-E三组中位OS期分别为未达到、（76.8±0.0）个月及（25.6±10.1）个月（三组总体*χ*^2^＝29.9，*P*<0.001；EM-B对non-EMD，*χ*^2^＝2.297，*P*＝0.130；EM-E对non-EMD，*χ*^2^＝35.332，*P*<0.001；EM-B对EM-E，*χ*^2^＝9.293，*P*＝0.002）（图1B）。生存曲线显示，无论是PFS还是OS，EM-B组患者和non-EMD组患者预后差异无统计学意义，EM-E组患者预后最差。

**图1:**
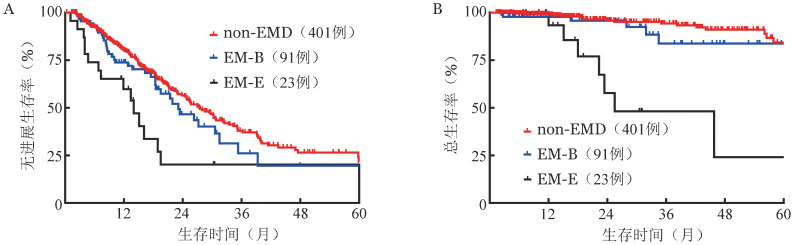
515例初诊多发性骨髓瘤患者non-EMD、EM-B和EM-E三组无进展生存（A）和总生存（B）曲线 注 non-EMD：非髓外病变；EM-B：骨旁髓外病变；EM-E：软组织髓外病变

3. NDMM患者PFS和OS的多因素分析：如表2所示，将年龄、性别、是否ASCT、细胞遗传学危险度、髓外MM类型、肌酐清除率和ISS分期纳入多因素分析，结果显示男性（*HR*＝1.511，*P*＝0.017）、接受ASCT（*HR*＝0.496，*P*＝0.001）、高危细胞遗传学（*HR*＝1.482，*P*＝0.037）和ISS Ⅲ期（*HR*＝1.539，*P*＝0.030）是PFS独立预后因素，而EM-E对PFS也有不良影响（*HR*＝1.874，*P*＝0.050）；接受ASCT（*HR*＝0.303，*P*＝0.036）、高危细胞遗传学（*HR*＝2.416，*P*＝0.039）、EM-E（*HR*＝8.779，*P*<0.001）是OS独立预后因素。将NDMM患者按是否接受ASCT进行亚组分析。23例EM-E患者中仅3例接受移植，因此EM-E未纳入亚组分析。接受ASCT的患者中，EM-B与non-EMD患者比较，无论是PFS（*P*＝0.884）还是OS（*P*＝0.420）差异均无统计学意义（图2）。在non-ASCT患者中，EM-B患者与non-EMD组比较，PFS差异无统计学意义（*P*＝0.095），但OS显著较差（*P*＝0.029）（图3）。

**表2:** 515例初诊多发性骨髓瘤患者整体人群无进展生存（PFS）和总生存（OS）的多因素分析

影响因素	PFS		OS	
	*HR*（95％*CI*）	*P*值	*HR*（95％*CI*）	*P*值
>65岁	1.010（0.699～1.460）	0.958	0.639（0.261～1.566）	0.328
男性	1.511（1.078～2.117）	0.017	1.249（0.526～2.967）	0.615
ASCT	0.496（0.327～0.754）	0.001	0.303（0.099～0.926）	0.036
高危细胞遗传学	1.482（1.024～2.144）	0.037	2.416（1.044～5.590）	0.039
non-EMD	参照		参照	
EM-B	1.414（0.939～2.131）	0.098	2.488（0.949～6.519）	0.064
EM-E	1.874（1.000～3.514）	0.050	8.779（2.927～26.338）	<0.001
肌酐清除率≤60 ml/min/1.73 m^2^	0.729（0.473～1.125）	0.154	2.351（0.839～6.585）	0.104
ISS Ⅲ期	1.539（1.042～2.273）	0.030	1.446（0.566～3.692）	0.441

注 ASCT：自体造血干细胞移植；non-EMD：非髓外病变；EM-B：骨旁髓外病变；EM-E：软组织髓外病变；ISS：国际分期系统

**图2:**
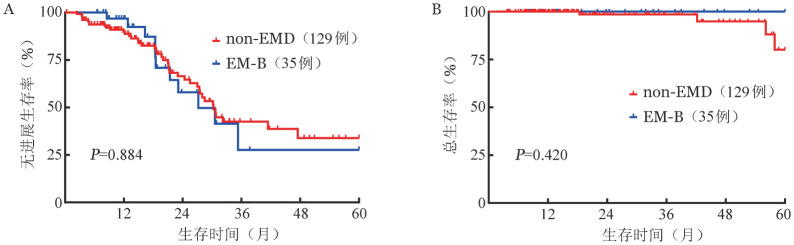
164例造血干细胞移植多发性骨髓瘤患者中non-EMD、EM-B两组无进展生存（A）和总生存（B）曲线 注 non-EMD：非髓外病变；EM-B：骨旁髓外病变

**图3:**
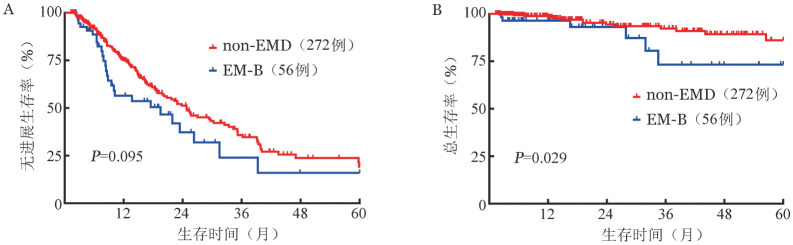
328例非造血干细胞移植多发性骨髓瘤患者中non-EMD、EM-B两组无进展生存（A）和总生存（B）曲线 注 non-EMD：非髓外病变；EM-B：骨旁髓外病变

4. 不同EM-B类型患者临床特征及预后：non-EMD、EM-B和EM-E组3年OS率分别为83％、76％和16％。进一步将EM-B组患者按照肿块大小分为3组：>1 cm并<3 cm（46例，51％）、≥3 cm并<5 cm（24例，26％）和≥5 cm（21例，23％），3年OS率分别为64％对78％对100％。将EM-B组患者按照肿块位置分为椎骨（34例，37％）、肋骨（29例，32％）、髂骨（11例，12％）、肩胛骨（6例，7％）和头颅骨（6例，7％）5组，3年OS分别为60％对78％对100％对100％对100％。将EM-B组按照无干预（72例，79％）和有干预（手术或放疗或手术联合放疗）（19例，21％）分为两组，3年OS率分别为84％对52％。

## 讨论

由于目前EMD定义尚不统一，有的文献仅指EM-E，而有的包括EM-B和EM-E但不区分两者[2]，或有的未纳入细胞遗传学资料[5]，因此EMD能否作为独立预后因素尚不清楚。本研究501例NDMM患者中114例伴EMD，占22％，与欧洲血液和骨髓移植学会登记的2014年初诊EMD比例（23.7％）相近[5]。本中心83％的EMD患者通过PET-CT检出，证实PET-CT等更加灵敏的影像学检测能增加初诊EMD检出率[6]。EM-B和EM-E的预后不同，本研究分别观察两者对NDMM患者预后影响。

本中心EM-E占NDMM患者4％，与国内外文献[5],[7]–[8]报道占比一致，EM-E患者预后差，中位OS时间只有25.6个月，与国内报道的22.5个月[8]和国外报道的19.2个月[9]接近。在纳入细胞遗传学危险度和是否接受ASCT等因素的预后多因素分析中，我们观察到EM-E是整体NDMM患者OS独立危险因素，符合既往研究[5],[9]报道。结果表明EM-B并非是PFS和OS的独立预后影响因素。从临床特征上观察，除了EM-E患者IgD比例较高外，三组患者之间差异均无统计学意义。EM-B和EM-E作为EMD的不同类型却预后不同，可能机制包括：（1）EM-E一般与高危细胞遗传学、高增殖性、凋亡逃避以及耐药有关[10]。国内有研究报道[8]28.6％EM-E患者髓内浆细胞17p缺失显著增高，我们也观察到17p缺失比例较高为24％。Billecke等[11]报道EM-E髓外浆细胞和EM-B相比，显示更高比例的t（4;14），17p缺失和MYC过表达，而且常表现和匹配患者髓内浆细胞不同的细胞遗传学，提示基因不稳定性导致EM-E预后不佳。（2）从转移途径分析，EM-B是骨髓瘤细胞突破骨皮质侵及周围连续性软组织在髓旁形成肿块，本质特征可能和髓内浆细胞接近；而EM-E是骨髓瘤细胞通过血源播散导致的远离骨髓原发灶的软组织或其他器官的病变[2]，提示EM-E侵袭程度更强。（3）从肿瘤微环境分析，Ryu等[12]通过单细胞转录组分析发现EM-E髓外病变的骨髓瘤细胞表达持续的MHC-Ⅰ类分子和上调表达低氧诱导因子-1-α（HIF1A），导致肿瘤微环境NK细胞和杀伤性T细胞功能受损，提示EM-E不良预后与肿瘤微环境的免疫逃逸有关。

EM-B患者临床更多见，我们发现EM-B大肿块（≥5 cm）并非预后不良，Vittorio等[13]也报道以3 cm为界的EM-B肿块之间预后差异无统计学意义。本研究发现椎骨和肋骨是最易受累的EM-B位置（分别为37％和32％），符合国外研究报道[13]。然而，椎骨受累EM-B 3年OS率仅60％，手术和（或）放疗干预EM-B患者的52％，且干预组所有死亡2例患者均发生在椎骨EM-B。临床观察中我们发现椎骨EM-B患者常伴下肢偏瘫，且易出现髓外复发，推测椎骨EM-B进展后易发生中枢侵犯或者远处转移，OS较差具体原因值得进一步探索。

我们发现在多因素分析中，无论是PFS还是OS，ASCT都是NDMM患者独立影响因素，显著改善预后。关于ASCT对EM-B作用，我们观察到在接受ASCT的患者中，与文献[5]报道一致，EM-B和non-EMD患者PFS和OS差异无统计学意义，而在non-ASCT患者中，EM-B的OS显著差于non-EMD（*P*＝0.029），而且PFS也呈现较差的趋势（*P*＝0.095），提示ASCT能改善甚至克服EM-B的不良预后。韩国Lee等[14]也发现EMD是non-ASCT患者群体OS的独立预后不良因素，该研究EMD主要包括EM-B。因此，对于不适合ASCT群体，EM-B患者不良预后仍需重视，联合包括硼替佐米和来那度胺等多种新药有可能改善这部分患者预后[2]。关于ASCT对EM-E作用，因本中心累计EM-E移植病例数较少，难以从目前资料得出ASCT对EM-E的作用。但Weinstock等[15]发现移植后初诊EM-E患者中位OS时间能达到4.1年，对比本中心数据，提示移植可能改善EM-E的不良预后。然而，Gagelmann等[5]报道即使在所有接受ASCT患者中进行多因素分析，EM-E仍是独立危险因素[5]，提示ASCT虽能改善但仍无法克服EM-E的不良预后（即达到和non-EMD患者相似预后）。对于二次移植，Gagelmann等[16]指出二次ASCT较单次能改善EMD预后，但是该研究也未作EM-B和EM-E的区分。新药临床试验很少纳入初诊EM-E的患者，而作为新兴疗法嵌合抗原受体T细胞（CAR-T细胞）对EM-E有效性目前局限于复发难治MM中继发EM-E的报道[17]–[19]，因此探索初诊EM-E的有效治疗策略任重道远。2021年英国血液学会专家共识对初诊适合ASCT的EM-E患者推荐在强化疗基础上联合二次移植甚至异基因造血干细胞移植，而对不适合ASCT的EM-E患者推荐包括硼替佐米、来那度胺和CD38单抗等多种新药联合方案[20]。

通过对114例NDMM伴EMD患者的总结，我们体会PET-CT的逐步普及将为我国初诊EMD患者基线资料和随访评估提供必要辅助手段。随着我国更多适合移植MM患者接受ASCT，将为克服EM-B患者不良预后以及改善EM-E患者生存提供保障。对于EM-E和不适合ASCT的EM-B这群高危NDMM患者，新药的早期或联合使用以及CAR-T细胞治疗前移能否改善预后，期待今后前瞻性临床研究结论解答。
